# Service readiness, health facility management practices, and delivery care utilization in five states of Nigeria: a cross-sectional analysis

**DOI:** 10.1186/s12884-016-1097-3

**Published:** 2016-10-06

**Authors:** Anastasia J. Gage, Onyebuchi Ilombu, Akanni Ibukun Akinyemi

**Affiliations:** 1 Department of Global Community Health and Behavioral Sciences, Tulane University, 1440 Canal Street, Suite 2200-22 Mail code: 8319, New Orleans, LA 70112-2824 USA; 2 Independent Researcher, 212 Loyola Avenue, Apartment 1105, New Orleans, LA 70112 USA; 3 Department of Demography and Social Statistics, Obafemi Awolowo University, Ile Ife, Nigeria

**Keywords:** Health services quality and management, Maternity services, Health facility delivery, Nigeria

## Abstract

**Background:**

Existing studies of delivery care in Nigeria have identified socioeconomic and cultural factors as the primary determinants of health facility delivery. However, no study has investigated the association between supply-side factors and health facility delivery. Our study analyzed the role of supply-side factors, particularly health facility readiness and management practices for provision of quality maternal health services.

**Methods:**

Using linked data from the 2005 and 2009 health facility and household surveys in the five states in which the Community Participation for Action in the Social Sector (COMPASS) project was implemented, indices of health service readiness and management were developed based on World Health Organization guidelines. Multilevel logistic regression models were run to determine the association between these indices and health facility delivery among 2710 women aged 15–49 years whose last child was born within the five years preceding the surveys and who lived in 51 COMPASS LGAs.

**Results:**

The health facility delivery rate increased from 25.4 % in 2005 to 44.1 % in 2009. Basic amenities for antenatal care provision, readiness to deliver basic emergency obstetric and newborn care, and management practices supportive of quality maternal health services were suboptimal in health facilities surveyed and did not change significantly between 2005 and 2009. The LGA mean index of basic amenities for antenatal care provision was more positively associated with the odds of health facility delivery in 2009 than in 2005, and in rural than in urban areas. The LGA mean index of management practices was associated with significantly lower odds of health facility delivery in rural than in urban areas. The LGA mean index of facility readiness to deliver basic emergency obstetric and neonatal care declined slightly from 5.16 in 2005 to 3.98 in 2009 and was unrelated to the odds of health facility delivery.

**Conclusion:**

Supply-side factors appeared to play a role in health facility delivery after controlling for socio-demographic factors. Improving uptake of delivery care would require greater attention to rural–urban inequities and health facility management practices, and to increasing the number of health facilities with fundamental elements for delivery of basic emergency obstetric and neonatal care.

**Electronic supplementary material:**

The online version of this article (doi:10.1186/s12884-016-1097-3) contains supplementary material, which is available to authorized users.

## Background

Although substantial progress has been made in reducing maternal mortality, the World Health Organization (WHO) estimated that in 2015, 303,000 women died from potentially avoidable problems in pregnancy or childbirth worldwide. Africa accounted for more than half of the global burden of maternal deaths, with women in the region having a 1 in 37 chance of dying in pregnancy/child birth compared to a 1 in 3400 chance in Europe – the largest difference between poor and rich countries on any health indicator. Nigeria, which constituted less than 1 % of the world’s population, accounted for 19 % of global maternal deaths and had an estimated maternal mortality ratio of 814 maternal deaths per 100,000 live births in 2015 [1]. Uptake of maternity care is low in Nigeria, with only 36 % of births occurring in a health facility (HF) and 38 % being assisted by a skilled provider [2].

Access to good quality obstetric care is critical for reducing maternal mortality. Globally, 15 % of pregnant women will experience obstetric complications, many of which are unpredictable, making the availability and quality of basic emergency obstetric and neonatal care (BEmONC) a public health imperative. While multiple factors may account for a high number of maternal deaths, empirical studies have suggested that poor quality services may lead to low coverage of maternal health (MH) care and non-effective and non-timely management of life-threatening complications of pregnancy/childbirth [3–7]. Existing evidence indicates that MH services in many parts of sub-Saharan Africa are deficient in terms of providing basic emergency obstetric care (BEmOC) [8, 9]. Some settings have been noted to lack essential drugs, reagents and equipment for MH care, high quality patient education, timely referral and transportation services, and an adequate number and mix of skilled providers [10, 11].

Studies of supply-side factors affecting the utilization of MH care services in Nigeria have revealed wide gaps in HF and provider performance. The health care system has been found to be suboptimal in terms of the availability of human resources and commodities at service delivery points [12]. However, evidence suggests that privately-owned facilities are better prepared to provide basic and comprehensive emergency obstetric care than public HFs [13]. Available evidence showed that the level of provision of almost all signal functions for basic and comprehensive emergency obstetric care was below the UN-recommended level while the direct obstetric case fatality rate, which is reflective of the quality of care, exceeded the United Nations-recommended maximum [14]. Of primary health care (PHC) facilities enrolled in the Midwives Service Scheme, a government program designed to address the national shortage of skilled birth attendants, 44 % did not provide all the components of ANC [15]. At the sub-national level, the availability and quality of life-saving obstetric services have been demonstrated to be poorer in northern compared to southern states, and in rural than in urban areas, a reflection of broader health system constraints [14].

Qualitative studies have revealed that barriers to HF delivery include cost, women’s concern that a companion will not be allowed to stay with them during labor, unfriendly attitude of health care providers, and concern about not being able to deliver in the preferred position [16]. Some studies have uncovered neglectful, abusive and disrespectful treatment of women during childbirth by healthcare providers, which tended to deter HF delivery [17–19]. Other factors identified by community members as affecting the quality of services delivered were inadequate trained health workers, shortage of essential drugs, and long distances to HFs [20].

Research has shown that at the individual-level, older, more educated, wealthier, urban and working Nigerian women are more like to deliver in a HF than their counterparts [21, 22], while those residing in the northern states are significantly less likely to deliver in a HF than those in southern states [23]. HF delivery has also been associated with Nigerian women’s participation in household decision making and attitudes regarding a wife’s ability to refuse sex [22, 24]; timing of initiation of antenatal care (ANC) and number of ANC visits at the primary health care center [21] and household levels [25]; and enrolment in a health insurance scheme [25]. Community-level determinants have included residence in areas with a high proportion of women who had secondary education and ethnic diversity, with the former factor being positively and the latter, negatively associated with HF delivery in Nigeria [23]. However, there is a lack of studies that have examined the extent to which structural aspects of health care are predictive of HF delivery or MH care utilization, more broadly, after controlling for individual-level factors.

This study contributed to filling this gap in the literature by examining the association between HF readiness to provide MH care and HF delivery in five states of Nigeria. The study is especially valuable since Nigeria’s contribution to the global burden of maternal deaths is one of the highest and since the country faces many health-system challenges, which are also found in other sub-Saharan African settings. Specific study objectives were to: (1) assess changes in HF readiness to deliver MH services over time; (2) examine the association with HF delivery of area-level indices of readiness to provide MH services and quality management practices at HFs; and (3) assess whether these associations changed significantly over time and were different in rural and urban areas. The results of the analysis could help improve understanding of the supply and demand nexus for HF delivery, which is critical for reducing deaths arising from complications of pregnancy.

## Methods

Data were drawn from the 2005 and 2009 surveys of the Community Participation for Action in the Social Sectors (COMPASS) project in Nigeria. The COMPASS Project was launched in 2004 with the aim of expanding participation, ownership and use of healthcare and education sector services at the community level in 51 local government areas (LGAs) across four states (Bauchi, Lagos, Kano and Nasarawa) and the Federal Capital Territory (FCT) of Nigeria over a period of 5 years. The project was designed with the aim of stimulating and promoting the integration of education and health development across all project activities at all levels.

The study was conducted in the 51 COMPASS LGAs and comprised three surveys: household, HF, and school. The household survey used a multi-stage stratified sampling design and collected information on reproductive and MH, child health, and HIV/AIDS-related knowledge and behaviors among women aged 15–49 and men aged 15–64 years. At the first stage of sampling, enumeration areas (EAs) were selected within each state, with probability proportional to the number of LGAs per state as follows: 1:1:2:2:1 for Bauchi, FCT, Kano, Lagos, and Nasarawa, respectively. At the second stage, 25 households were selected within each sample EA using systematic random sampling. Fieldwork was conducted from July-August 2005 and from mid-June to August, 2009.

The HF surveys (including comprehensive health care centers; public PHC centers; health, maternity, private, or uniformed services clinics; health posts; and dispensaries and patent medicine vendors (PMVs)) were implemented at the same time as the household surveys. The HF survey was census of all public and primary HFs and PMVs serving the households surveyed. Consequently, the HF survey included some service delivery points that were located outside of the sample EAs and LGAs selected for the household survey. A total of 233 and 286 HFs were surveyed and facility inventories and provider interviews conducted in 2005 and 2009, respectively. The LGA was used to link the HF and household data.

The outcome was binary and indicated whether the most recent birth in the past 5 years was delivered in a medical institution. The first two indices of service readiness were constructed based on World Health Organization guidelines on tracer elements for assessment of general service readiness [26]. The third index measured management practices supportive of quality MH services. Each component of the index was binary unless otherwise indicated.

1. Adapted Index of basic amenities for the provision of MH services:
  This was a 7-item additive index measuring the presence of the following resources in HFs: power (a grid or a functional generator and fuel for it); a protected water source; communication equipment (a working phone or shortwave radio); access to an incinerator for disposal of potentially contaminated waste and items that are not reused such as bandages and syringes; HF assessed to be clean; public transportation within 1 kilometer; and beds for overnight stay. Unfortunately, the HF questionnaire excluded four recommended components – access to computer with email/internet, access to adequate sanitation facilities, availability of emergency transportation, and availability of a room with auditory and visual privacy for patient consultations. The resulting HF index ranged from 0 to 7 and had a Cronbach’s alpha of 0.612 in 2005 and 0.793 in 2009. The LGA-based measure was the mean adapted index of basic amenities per HF surveyed in the LGA.
2. Adapted Index of readiness to deliver BEmONC:
  Components of the index were based on WHO (2010) recommendations and covered staff training, equipment and medicines/commodities, and included: availability of guidelines for delivery; staff trained; emergency transportation not considered problematic; examination light; suction apparatus/mucous extractor; vacuum aspirator or dilation and curettage kit; newborn bag and mask; partograph; clean gloves; injectable uterotonic; injectable antibiotic; and intravenous solution with infusion set. Data were not collected on three recommended components: manual vacuum extractor, antibiotic eye ointment for the newborn, and magnesium sulphate. HFs that did not provide delivery/newborn care were assigned the value “0” on this indicator. The resulting 22-item additive index represented the cumulative availability of components required to provide BEmONC, had a Cronbach’s alpha of 0.925 in 2005 and 0.939 in 2009, and ranged from 0 to 21 for HFs in the sample. The LGA-based measure was the unweighted average number of items present in the HFs that provided delivery and newborn care in the LGA.

### Index of management practices supportive of quality MH services

This index measured the routine use of quality assurance methods by the HF; the occurrence and content of supervisory visits in the past six months; the availability of systems for client feedback; the presence of up-to-date client and birth registers; and the availability of a skilled provider. Questions on use of quality assurance methods asked non-PMVs whether any of the following methods of quality assurance were routinely used by the facility: (a) supervisory checklist for health system components (e.g., service-specific equipment, medications and records) based on standard and protocol; (b) supervisory checklist for health service provision (e.g., observation checklist) based on standards and protocol; (c) system for identifying and addressing quality of care that is implemented by staff or specific service level; (d) facility-wide review of mortality; (e) periodic audit of medical records or service registers; (f) quality assurance committee/team; (g) regional/district health management teams; and (h) other method. Components pertaining to supervision were asked separately for ANC/postpartum care and delivery/newborn care and measured (h) number of times in the last six months the provider’s delivery/newborn care was supervised and for the most recent supervisory visit, whether the supervisor (i) checked the provider’s records/reports; (j) observed his/her work; (k) provided feedback on his/her performance; (l) provided updates on administrative or technical issues related to his/her work; (m) discussed problems the provider had encountered; (n) discussed job expectations; and (n) anything else. Components pertaining to the availability of systems for client feedback asked whether the HF had the following systems for determining client opinion about the HF or its services: (o) suggestion box; (p) client survey form; (q) client interview; (r) other system. The variable measuring up-to-date birth registers consisted of four categories: no register, register not seen, register seen – last entry more than 7 days ago, and register seen – entry in past 7 days. One component of the index measured whether a skilled birth attendant (doctor, nurse or midwife) was present at the facility or on call 24 h a day, including weekends to provide delivery care and their actual involvement in conducting deliveries. This variable was coded as follows: 4 if a skilled attendant was present and always conducted deliveries; 3 if a skilled attendant was present but deliveries were sometimes conducted by primary- or auxiliary-level staff; 2 if a skilled attendant was on call and always conducted deliveries; 1 if a skilled attendant was on call but deliveries were sometimes conducted by primary or auxiliary level staff; and 0 if a skilled attendant was not present or on call 24 h a day, including weekends, to provide delivery care. The resulting 28-item HF index ranged from 0 to 31, with a Cronbach’s alpha of 0.8834 in 2005 and 0.895 in 2009. We calculated the LGA-level mean index based on the scores of all non-PMVs that provided MH services in the LGA.

The analysis controlled for the following individual-level variables: year of survey (2009 versus 2005); duration of residence in the area (years); age as reported; number of children ever born; education (none, primary, secondary/higher); marital status (married, living with a partner, not in union); type of place of residence (urban, semi-urban, or rural); state (Bauchi, Kano, FCT, Lagos, Nasarawa); counseling about pregnancy complications (no ANC from a health professional, not counseled, counseled about pregnancy complications and where to go); and household wealth (low, medium, high). Household wealth represented by tertile of an index constructed from the household ownership of the following amenities/items, using principal components analysis: refrigerator, electricity, piped water, flush toilet, bicycle, motorcycle, car, television, radio, and telephone/cellular phone). The index was based on the first component, which explained 44.2 % of the common variances of all ten components. Scree plot inspection revealed a distinct one-factor solution. The Kaiser-Meyer Olkin measure of sampling adequacy was 0.867.

The analysis was based on women whose most recent birth occurred in the five years preceding the survey. Descriptive statistics were calculated for all variables of interest. We computed *F*-tests to investigate the association between the HF delivery rate and LGA measures of service readiness, taking into consideration the multi-stage sampling design. Two-level random-intercept logistic regression models that offered simultaneous consideration of *i* women (Level 1) nested in *j* LGAs (Level 2) were estimated to take into consideration the hierarchical clustered structure of the data, which if ignored, could generate improper standard errors, and to incorporate random effects at the LGA and individual levels to account for unobserved factors. Adjusted odds ratios (AORs) and 95 % confidence intervals (CIs) were estimated from regression statistics using the generalized latent and mixed model command in Stata 12.1.0.^15^ Variance inflation factors (VIFs) suggested that multicollinearity was not a major concern: the mean VIF was 1.82 and the highest was 2.80. Intra-class correlation coefficients (ICC) were used to evaluate how the odds of HF delivery varied between LGAs and were calculated as:

$$ \rho = \left({\upsigma^2}_{\upmu}/\ \left({\upsigma^2}_{\upmu} + {\uppi}^2/3\right)\right) $$

where σ^2^_μ_ is the intercept variance and π^2^/3 = 3.29 and represents the level-1 residual variance for a logit model. The analytical sample consisted of 51 LGAs and 2710 mothers whose last birth occurred in the past five years and who had no missing data on variables of interest. No significant differences between missing and non-missing cases were detected.

## Results

### Women’s characteristics

Table 1 shows significant differences between 2005 and 2009 in socio-demographic characteristics of women who gave birth in the past 5 years. Women interviewed in 2005 sample had stayed in their communities for 5 more years on average, were slightly younger, and had given birth to one less child on average than those interviewed in 2009. Slightly more than a third of women had no formal schooling but more women with secondary/higher education were interviewed in 2005 (43.02 %) than in 2009 (31.46 %). More than half of the sample was married but the percentage of women who were not in union was half as high in 2009 as in 2005.

**Table 1:** Characteristics of women whose last child was born in the past five years and of local government areas surveyed, Nigeria 2005 and 2009

	2005		2009	
Characteristics	Mean/Percent	*N*	Mean/Percent	*N*
Individual level				
*Mean (SE)*				
Duration of residence***	14.987 (.356)	1338	9.994 (.281)	1372
Age in years***	31.524 (.308)	1338	33.18 (.251)	1372
Number of children ever born***	2.126 (.064)	1338	3.26 (.060)	1372
*Percent distribution*				
Educational attainment***				
None	37.81 %	549	33.51 %	513
Primary	19.17 %	291	35.03 %	471
Secondary/higher	43.02 %	498	31.46 %	388
Marital status				
Currently married	59.66 %	842	70.93 %	975
Living with a partner	18.58 %	225	19.96 %	282
Not in union	21.76 %	271	9.11 %	115
Counseling about pregnancy complications				
No ANC from HP	62.25	822	35.83	500
ANC from HP, no counseling	14.47	191	20.97	265
ANC from HP, counseling	23.28	325	43.19	607
Type of place of residence***				
Urban	60.20 %	634	60.97 %	604
Semi-urban	6.22 %	112	13.32 %	267
Rural	33.58 %	592	25.71 %	501
State*				
Bauchi	16.30 %	211	13.06 %	166
Kano	25.71 %	365	24.64 %	342
Federal Capital Territory	3.64 %	171	4.99 %	231
Lagos	48.55 %	387	50.43 %	395
Nasarawa	5.79 %	204	6.88 %	238
Household wealth***				
Low	31.52 %	525	26.28 %	466
Medium	25.18 %	369	42.64 %	522
High	43.31 %	444	31.08 %	384
Total	100.00 %	1338	100.00 %	1372
HF level				
Mean index of basic amenities for the provision of MH care +	2.48 [1.49]		2.79 [2.05]	
Mean index of readiness to deliver BEmONC +	5.05 [5.63]		4.20 [5.62]	
Mean index of management practices supportive of quality MH care	6.52 [7.71]		5.71 [7.57]	
Number of HFs	233		286	
LGA level				
Mean index of basic amenities for the provision of MH care	2.21 [0.88]		2.51 [0.94]	
Mean index of readiness to deliver BEmONC +	5.16 [3.26]		3.98 [3.21]	
Mean index of management practices supportive of quality MH care	6.66 [5.86]		6.22 [4.40]	
Number of LGAs	44		45	

Data are weighted at the individual level. Significance levels pertain to change in characteristics between 2005 and 2009

*HP* Health care provider, *SE* Standard error, [] Standard deviation

+*p* < .10; *p < .05; ***p < .001

There were also significant differences in the distribution of the sample by type of place of residence and household wealth. More rural women were interviewed in 2005 than in 2009 (33.58 % versus 25.71 %) and a lower proportion lived in wealthy households in 2009 than in 2005 (31.08 % versus 43.31 %). There was a marked increase in the percentage of women who reported being counseled about warning signs of pregnancy complications during ANC visits between 2005 and 2009, which corresponded with a substantial reduction in the percentage of women who did not seek ANC from a health professional.

### Service readiness

On average, readiness to deliver MH services was suboptimal at the HF and LGA levels in 2005 and showed little improvement by 2009. As Table 1 shows, on average, facilities had about 2.97 (SD = 2.05) basic amenities for ANC provision and 4.2 (SD = 5.62) of the components for delivery of BEmONC, with a management index of 5.71 out of a maximum score of 31 in 2009. The availability of individual components of health service readiness and quality management practices at the HFs surveyed is shown in Additional file 1: Table S1. Overall, 36.71 % of HFs surveyed in 2009 were found to be providing some form of delivery and newborn care, compared to 51.50 % in 2005, a statistically significant difference that was similarly observed for ANC provision (40.56 % in 2009 versus 64.81 % in 2005). With the exception of a protected water source, fewer than half of the HFs had basic amenities for the provision of ANC. The availability of electricity always/often or a functional generator and fuel for it declined significantly from 2005 to 2009 (from 58.80 to 39.16 %). Less than one in five HFs had a working phone or shortwave radio and fewer than half had beds for overnight stay or were assessed to be clean. Less than 10 % of HFs disposed of contaminated waste by burning in an incinerator. Overall, the average percentage of HFs with all basic amenities measured was 1.75 % in 2009 and none in 2005 (not shown).

Concerning the availability of components required for delivery of BEmONC, Additional file 1: Table S1 shows the availability of tracer items at HFs surveyed, including trained staff, guidelines, equipment, and medicines and commodities. Note that the tabulations pertained to HFs that offered delivery and newborn care unless otherwise indicated. In 2009, the average guideline availability was 39.05 % for delivery care and 28.57 % for the partograph, and showed little change since 2005. Fewer than two out of three HFs had at least one staff who had ever received in-service training on topics relevant to MH care. The most common training topic was exclusive breastfeeding, (60.95 %) and the least common was the partograph (32.38 %). Management of high-risk pregnancies was the only topic for which there was a significant increase over time in the availability of staff who had ever received in-service training (from 38.33 % in 2005 to 55.24 % in 2009).

The least commonly available pieces of equipment/supply for delivery of BEmONC were manual vacuum aspirators and dilation and curettage kits for removing retained products of conception (about 30 %). At least three out of four HFs surveyed had injectable uterotonic and clean gloves for delivery. Mucous extractors and a 24-h functioning light source (including a lantern) for delivery were present in about three out of five HFs surveyed. In 2009, injectable antibiotics were observed in roughly one out of every three and intravenous solution in about half of HFs surveyed (36.71 and 51.43 %, respectively), with little change since 2005.

Almost two out of three HFs surveyed in 2009 had a routine program for monitoring the quality of care. Less than two out of five had a system for determining client opinion about the HF or its services, a decrease from 2005 (38.33 % versus 29.18 %, not shown). Supervision content was less than optimal for both antenatal and delivery/newborn care (see Additional file 1: Table S1). More than half of HFs surveyed did not participate in regular reviews of maternal or newborn deaths or near miss deaths. However, there was a significant increase in the percentage of HFs that conducted such reviews for mothers only and a corresponding decline in the percentage conducting such reviews for newborns only. Less than half of HFs that provided ANC or delivery/newborn care had up-to-date registers. Between 2005 and 2009, there was a significant decrease in the percentage of HFs that had no skilled attendant (doctor nurse or midwife) at the facility or on call 24 h a day including weekends, to provide delivery care. While the percentage of HFs with a skilled attendant present was about 41 % in both surveys, there was a decrease over time in the percentage that had an auxiliary-level health care provider on call and an increase in the percentage that had a doctor, nurse or midwife on call.

The LGA mean adapted index of basic amenities for the provision of ANC was 2.205 (SD = 0.878) in 2005 and 2.511 (SD = 0.944) in 2009, a statistically insignificant change. The LGA mean adapted index of readiness to deliver BEmONC was low in 2005 and declined to 3.978 (SD = 3.209) in 2009 (*p* < .10). A similar decrease was observed at the HF level. There was very little change over time in the LGA mean index of management practices supportive of quality MH care (see Table 1).

### Bivariate results

For the bivariate analysis, each LGA-based index was operationalized into a binary variable measuring whether a given LGA was at/above or below the median value of the index. As Table 2 shows, 44.14 % of women delivered their most recent birth in a HF in 2009, compared with 25.41 % in 2005. There were significant differentials in HF delivery by type of place of residence, with the rate being 4–5 times higher among urban than rural women (e.g., 63.35 % versus 13.15 % in 2009). Contrary to expectations, the HF delivery rate tended to be higher among rural and semi-urban women who lived in LGAs with low (below the median) as compared to high (at/above the median) levels of readiness to provide MH services. However, for urban women, the HF delivery rate in 2009 was higher among those residing in LGAs that were at/above as opposed to below the median indices of basic amenities, readiness to deliver BEmONC, and management practices supportive of quality MH care. For example, in 2009, 50.34 % of urban women who resided in LGAs that ranked below the median index of management practices gave birth in a HF compared to 62.37 % of their counterparts who resided in LGAs that were at/above the median of the index.

**Table 2:** HF delivery rate among women whose last child was born in the past five years by LGA characteristics and type of place of residence, Nigeria 2005 and 2009

LGA characteristics	Urban	Semi-Urban	Rural	Total
	2005			
Index of basic amenities for the provision of ANC		*		
Below median	42.60 %	(58.93 %)	4.36 %	25.91 %
At/above median	33.91 %	34.05 %	5.88 %	25.29 %
Index of readiness to deliver BEmONC		*		
Below median	38.76 %	(56.81 %)	4.01 %	28.35 %
At/above median	33.86 %	32.22 %	6.03 %	24.29 %
Index of management practices supportive of quality MH care	*	*		
Below median	32.47 %	46.50 %	7.33 %	27.97 %
At/above median	39.63 %	28.43 %	4.62 %	22.78 %
Total	35.27 %	37.46 %	5.49 %	25.41 %
Total number of women	634	112	592	1338
	2009			
Index of basic amenities for the provision of ANC	*			
Below median	50.01 %	(20.08 %)	19.44 %	43.01 %
At/above median	61.31 %	36.34 %	14.68 %	44.42 %
Index of readiness to deliver BEmONC		*	**	**
Below median	56.34 %	42.09 %	23.05 %	51.25 %
At/above median	63.35 %	25.07 %	13.18 %	35.58 %
Index of management practices supportive of quality MH care	**		*	
Below median	50.34 %	35.84 %	19.77 %	40.38 %
At/above median	62.37 %	32.63 %	13.15 %	46.11 %
Total	58.51 %	34.25 %	15.21 %	44.14 %
Total number of women	604	267	501	1372

Data are weighted

*p < .05; **p < .01

() Less than 25 cases

### Multivariate results

Table 3 shows AORs, CIs, and parameters from two-level logit regression models of HF delivery. The regression analysis was done in several stages. We started by estimating a null model (intercept-only) model that permitted a partitioning of the total variance into two variance components. Using estimates from the null model (with just a multilevel constant term, the LGA-specific random effect and no explanatory variables), we obtained an intra-class correlation coefficient of 0.327, which implied that more than a third of the explained variance in HF delivery was attributable to LGA-level variables.

**Table 3:** Results of multilevel models of HF delivery of last births in the past five years, Nigeria 2005 and 2009

	Model 1		Model 2	
Variables	AOR	95 % CI	AOR	95 % CI
*Individual Level*				
Year of survey				
2005	1.000		1.000	
2009	0.473	(0.185, 1.208)	1.180	(0.832, 1.674)
Duration of residence (years)	0.997	(0.985, 1.010)	0.998	(0.985, 1.010)
Age (years)	1.002	(0.986, 1.018)	1.000	(0.985, 1.017)
Education				
None	1.000		1.000	
Primary	1.331	(0.978, 1.812)	1.285	(0.944, 1.750)
Secondary/higher	1.577**	(1.120, 2.219)	1.576**	(1.120, 2.218)
Marital Status				
Currently married	1.000		1.000	
Living with partner	0.921	(0.675, 1.259)	0.918	(0.673, 1.256)
Not in union	0.828	(0.557, 1.231)	0.817	(0.551, 1.213)
No. of children ever born	0.982	(0.924, 1.043)	0.978	(0.920, 1.040)
Type of place of residence				
Urban	1.000		1.000	
Semi-urban	0.614*	(0.401, 0.939)	0.457	(0.126, 1.664)
Rural	0.440***	(0.281, 0.690)	0.330*	(0.128, 0.849)
Counseled about pregnancy complications				
No ANC from HP	0.021***	(0.014, 0.033)	0.022***	(0.014, 0.033)
ANC from HP, not counseled	1.000		1.000	
ANC from HP, counseled	1.520**	(1.157, 1.997)	1.529**	(1.163, 2.011)
Household wealth				
Low	1.000		1.000	
Medium	1.586*	(1.102, 2.284)	1.547*	(1.080, 2.218)
High	2.054***	(1.347, 3.131)	2.050***	(1.347, 3.119)
*LGA-level*				
Index of basic amenities for ANC provision	0.732	(0.529, 1.103)	0.892	(0.704, 1.131)
Index of readiness to deliver BEmONC	0.987	(0.915, 1.065)	1.002	(0.938, 1.069)
Index of management practices supportive of quality MH care	1.048	(0.983, 1.117)	1.045*	(1.001, 1.091)
State				
Lagos	1.000		1.000	
Bauchi	0.250***	(0.116, 0.542)	0.303**	(0.144, 0.637)
Kano	0.297***	(0.168, 0.526)	0.286***	(0.165, 0.493)
Federal Capital Territory	1.124	(0.573, 2.207)	1.224	(0.641, 2.334)
Nasarawa	0.578	(0.294, 1.135)	0.643	(0.338, 1.223)
*Interaction Terms*				
Index of basic amenities x 2009	1.692*	(1.110, 2.581)		
Index of readiness to deliver BEmONC x 2009	1.052	(0.936, 1.181)		
Index of management practices x 2009	0.948	(0.874, 1.028)		
Index of basic amenities x semi-urban			1.269	(0.764, 2.108)
Index of basic amenities x rural			1.536*	(1.058, 2.231)
Index of management practices x semi-urban			0.975	(0.920, 1.032)
Index of management practices x rural			0.924**	(0.875, 0.976)
Constant	1.989	(0.739, 5.348)	1.221	(0.459, 3.249)
*LGA Random Term*				
Variance (covariance)	0.200 (0.094)		0.165 (0.083)	
Number of LGAs	51		51	
Number of women	2710			

*AOR* Adjusted odds ratio, *CI* Confidence interval

*p < .05 **p < .01; ***p < .001

Model 1 of Table 3 was the full model with an interaction term between year of the survey and each of the LGA measures of service readiness and revealed that the LGA mean adapted index of basic amenities for the provision of MH care was more positively associated with the odds of HF delivery in 2009 than in 2005 (*p* < .05). Model 2 included interaction terms between the LGA measures of service readiness and type of place of residence. This model showed that the LGA mean adapted index of basic amenities was more positively associated (AOR = 1.536, 95 % CI = 1.058, 2.231) and the index of management practices more negatively associated with the odds of HF delivery among rural women compared to urban women (AOR = 0.924; 95 % CI = 0.875, 0.976). We also tested the associations between readiness to deliver ANC services and the odds of HF delivery (not shown). Those associations were statistically insignificant and were omitted, therefore, from the final models.

The likelihood ratio test was applied to test the significance of the random intercept by calculating the difference in the observed deviances between the full model without interaction terms and a simple logistic regression model with the same explanatory variables. A deviance difference of 13.257 (1° of freedom) was obtained, implying that there were statistically significant differences between LGAs in the odds of HF delivery.

At the individual level, the regression results highlighted the role of ANC and counseling on pregnancy complications in predicting HF delivery. Women who did not seek ANC from a health professional were significantly less likely than those who did but received no counseling on warning signs of pregnancy complications to give birth in a HF (AOR = 0.021; *p* < .001). Compared to the latter group of women, those who sought ANC from a health professional and were counseled on warning signs of pregnancy complications and where to go for treatment had significantly higher odds of HF delivery. Socioeconomic determinants of HF delivery included education and household wealth. The odds of HF delivery were at least one and a half times as high among women with secondary or higher education as among those with no education (AOR = 1.577; 95 % CI = 1.120, 2.219 in Model 1). Household wealth was positively associated with the odds of HF delivery, with women from the wealthiest households having twice the odds of HF delivery of those from the poorest households (see Table 2).

## Discussion

The purpose of this study was to examine how readiness to deliver MH services was associated with HF delivery, while taking into consideration underlying socio-economic determinants of health service use, such as education and household wealth. The study also tried to detect changes in measures of health service readiness over time and modelled some of the dynamic interactions that potentially existed between those aspects of service delivery and type of place of residence.

The analysis uncovered critical gaps in service readiness and HF capacity to provide BEmONC and corroborated national and subnational studies of HFs in Nigeria which showed major deficiencies in the health care system. One national study had found that most PHC facilities had no functional equipment for maternal and child health services while another had showed that 44 % of PHCs enrolled in the Midwives Service Scheme, an initiative implemented by the Federal Government of Nigeria to address the shortage of skilled birth attendants in rural areas, did not provide all components of MH care [15]. In a LGA of Southwest Nigeria, none of the facilities met the criteria for a BEmOC facility, 46 % were unmanned by unskilled health attendants, and none of the health workers in the LGA had ever been trained in lifesaving skills. In addition, there was a widespread lack of BEmONC equipment and supplies [27]. Similarly a rapid assessment of 121 PHCs revealed that most were unable to provide all BEmOC services and generally lacked clinical staff needed to dispense maternal and neonatal care services, ambulances, and uninterrupted electricity supply whenever there were obstetric emergencies. Although secondary HFs scored higher on these services, like PHCs, they tended to lack infrastructure for neonatal care [28]. Similarly, Abegunde et al. found that in Bauchi state, which was also included in the present study, the availability, utilization, and quality of EmOC services were suboptimal [14].

Although the rate of HF delivery increased significantly from 2005 to 2009, more than half of women in the sample delivered outside of HFs. The regression analysis revealed that the adapted index of basic amenities for the provision of ANC was more positively associated with the odds of HF delivery in 2009 than in 2005, and in rural than urban areas. While assessing and defining the quality of care can be difficult, research suggests that for poor and vulnerable clients, the most important dimensions of quality tend to include facility amenities, the others being technical competence, interpersonal relations, and accessibility [20]. Good infrastructure and hygiene, which were captured by our adapted index of basic amenities, may have been related to greater client satisfaction with the physical environment of the HF, and served as a greater catalyst for delivery care utilization among rural as compared to urban women.

We also found that the index of management practices supportive of quality MH care was associated with significantly lower odds of HF delivery among rural women compared to their urban counterparts. This index reflected quality assurance and supportive management practices, which together with infrastructural decay, have been described as “the bane of efficient PHC delivery” in Nigeria [29]. Given the insufficient quantity of care (even of lower-level HFs) in rural areas and rural women’s’ limited choice of HFs, these results are to be expected.

Surprisingly, readiness to deliver BEmONC was unrelated to the odds of HF delivery, after controlling for other factors, and called attention to sociocultural and other explanations for the utilization of MH services, which were not captured by our model. These explanations include transportation difficulties; attitudes of health workers; affordability, especially if supply constraints lead to women being required to bring their own supplies such as gloves; lack of privacy; women’s perception of being in good health; gender norms that constrain women’s mobility; spouses and relative’s disapproval of institutional delivery; and traditional beliefs and practices [16, 17, 22, 28, 30].

The limited improvements in components of health service readiness may be due to several factors, such as lack of political will and inadequate resource allocation to health system strengthening at the national and local levels over the years. However, the Federal Government of Nigeria is implementing several initiatives to improve the availability of, access to, and quality of MH services and address rural–urban inequities in service provision. The maternal and child care component of the Federal Government of Nigeria’s Subsidy Reinvestment Program has identified 125 general hospitals across 36 states and 500 out of the 23,000 frontline PHC facilities for refurbishment, upgrading, equipping, supply of drugs, and the employment and deployment of skilled health workers. The implementation of the Midwives Service Scheme (MSS) is addressing the shortage of skilled birth attendants at the primary healthcare (PHC) level, particularly in rural areas. Conditional cash transfers are being provided to pregnant women to address the indirect costs of care seeking, which partially contribute to the low demand for ANC and delivery services [31]. Such programs must be scaled up and accompanied by regular supportive supervision in order to improve the utilization of MH services and save lives.

The dearth of research on supply-side determinants of MH care utilization precluded the specification of hypotheses about the importance of service readiness as a predictor of HF and a nuanced comparison of our findings with those of other studies. Although the evidence is inconsistent, the effects often small in magnitude, and causality a concern, a number of studies have found a significant association between elements of HF readiness and family planning (FP) outcomes, even after controlling for individual-level factors. Using a composite measure of infrastructure and facility readiness to provide FP services, Hong, Montana and Mishra found that measures related to counseling and the examination room had significant positive effects on IUD use in Egypt [32]. Similarly, using an index score of the service delivery infrastructure, medical equipment, essential medicines, number of contraceptive methods available on the day of the visit and the number of staff trained in FP, Do and Keonig found that residence in communes with higher quality health centers was associated with significantly lower risk of method discontinuation [33]. However, other studies have found a weak or non-significant link between HF readiness and client contraceptive behavior [34, 35].

The analysis presented here focused on only one of the WHO-recommended five domains of general service readiness – basic amenities – with some adaptation. At earlier stages of the analysis, we examined two other domains, notably basic equipment and standard precautions for prevention of infection, in the regressions but these domains were unrelated to the odds of institution delivery, and their associations did not vary significantly over time or by type of place of residence. Consequently, they were omitted from the final regression models. No data were collected on laboratory tests (hemoglobin, blood glucose, HIV rapid diagnostic tests (RDT), syphilis RDT, malaria RDT or smear, TB microscopy, general microscopy, urine pregnancy test, urine dipstick), precluding us from including the laboratory domain of general service readiness in our analysis. Due to survey’s focus on maternal and child health and HIV/AIDS, data were not collected on medicines for non-communicable diseases (Salbutamol, Glibenclamide, Atenolol, Captopril, Simvasatin, Amitriptyline, Diazepam, Omeprazole), on one medicine for infectious diseases (Ceftriazone), and on one pain medication (Diclofenac). As data were available for only four of the 14 essential medicines (Ciprofloxacin, Co‐trimoxazole, Amoxicillin, and Paracetamol), a decision was made to exclude the essential-medicines index from the analysis.

Due to lack of data, the adapted index of basic amenities could not include four recommended components: access to a computer with email/internet; access to adequate sanitation facilities; availability of emergency transportation; and availability of a room with auditory and visual privacy for patient consultations. The addition of those components could have increased the reliability of the index given that the more items there are in an index that is designed to measure a particular concept, the more reliable will be the index. It is to be noted that Cronbach’s alpha for the adapted index of basic amenities was much lower in 2005 (0.612) than in 2009 (0.793), which signified poorer consistency of the item responses in the earlier survey. One factor that contributes to the consistency of an index is stable characteristics of the attribute being measured but between 2005 and 2009, several interventions implemented by development partners such as COMPASS and PATH targeted the provision and improvement of basic amenities in HFs, among other things. While the adapted index of basic amenities enabled us to have one statistical measure with which to gauge the availability of basic amenities in HFs serving COMPASS LGAs, the low reliability of the adapted index of basic amenities in 2005 increased the risk that our analysis underestimated or failed to detect the true association between the adapted index and the odds of HF delivery in 2005. This limitation should be borne in mind in the interpretation of the findings.

The study raised important methodological issues regarding how best to link population and HF data. Although the survey identified the universe of HFs actually used by households, the administrative boundaries of the LGA were artificially imposed on the data in order to identify level-two units for the multilevel regression and administrative areas for a potential programmatic response. The question arose as to whether the sampled HFs that fell within a given LGA characterized well where residents were obtaining MH care. There were two caveats. First, many individuals did not use HFs for MH care, which likely introduced biases in the sample. Second, our methodological approach combined two concepts: “Where LGA residents could go” and “where LGA residents did go” for health services. In the survey, GPS coordinates were obtained for HFs but not for households or EAs, an unfortunate omission. In addition, ensuring that there were enough facilities surveyed and service providers interviewed in areas that were closer approximations of neighborhoods than the LGA was a challenge and influenced how level-two units were defined.

Other limitations of the data stemmed from their cross-sectional nature, making it difficult to establish causality. Endogeneity was a concern as MH services may have been placed in areas with higher demand and fertility levels, potentially leading to an overstatement of the results. Temporal ordering was also of concern as measures of the MH service delivery environment may not have preceded delivery. Other limitations were that the data were not representative of the states surveyed and that we were unable to measure the quality of the services provided. Whether a provider carries out the right actions and the extent to which this translates into the right actions by the patient are best captured through exit interviews, provider-patient observations, mystery client studies, and follow-up studies of patients. These methods of data collection were not included in the HF survey. Finally, the odds of HF delivery may be determined by unobserved factors such as cultural beliefs surrounding delivery, transportation networks, financial costs of care, and provider shortage and absenteeism. Future research should explore these issues.

## Conclusions

The findings call for interventions that target specific elements of MH service delivery. Improving the uptake of delivery care would require greater attention to rural–urban inequities and HF management practices, and to increasing the number of HFs with fundamental elements for delivery of BEmONC. As this study focused on the structural aspects of the MH services and a limited number of sociodemographic factors, research is needed to elucidate the linkages between the social-psychological aspects of care and the utilization of MH services.

## Additional file

Additional file 1: Table S1. HF availability of components of service readiness and quality management, Nigeria 2005 and 2009. (DOCX 27 kb)

## Abbreviations

AIDS: Acquired immune deficiency syndrome
ANC: Antenatal care
AOR: Adjusted odds ratios
BEmOC: Basic emergency obstetric care
BEmONC: Basic emergency obstetric and newborn care
CI: Confidence interval
COMPASS: Community Participation for Action in the Social Sector
EA: Enumeration area
FCT: Federal Capital Territory
FP: Family planning
HF: HF
HIV: Human immune deficiency virus
ICC: Intra-class correlation coefficient
LGA: Local government area
MH: Maternal health
PHC: Primary health care
PMV: Patent medicine vendor
RDT: Rapid diagnostic test
SD: Standard deviation
SE: Standard error
VIF: Variance inflation factor
WHO: World Health Organization
