# Supplementary material for: Service readiness, health facility management practices, and delivery care utilization in five states of Nigeria: a cross-sectional analysis
Source: BMC Pregnancy Childbirth. 2016 Oct 6;16:297. doi: 10.1186/s12884-016-1097-3 (PMC5054586; doi:10.1186/s12884-016-1097-3)
Supplement: Additional file 1: Table S1. — HF availability of components of service readiness and quality management, Nigeria 2005 and 2009. (DOCX 27 kb) [file 12884_2016_1097_MOESM1_ESM.docx]

Appendix Table 1 HF availability of components of service readiness and quality management, Nigeria 2005 and 2009

| **Component** | **2005** | **2009** | ***p*-value** |
| --- | --- | --- | --- |
| *Services provided* |  |  |  |
| ANC | 64.8 | 40.6 | <0.001 |
| Delivery and newborn care | 51.5 | 36.7 | <0.001 |
| *Index of basic amenities for provision of ANC* (a) |  |  |  |
| Electricity always/often or functional generator and fuel for it | 58.8 | 39.2 | 0.000 |
| Working phone/shortwave radio | 16.3 | 17.5 | 0.723 |
| Protected water source | 82.4 | 79.7 | 0.428 |
| Disposes of contaminated waste by burning in incinerator | 7.3 | 6.6 | 0.771 |
| HF assessed to be clean | 44.2 | 47.9 | 0.401 |
| HF is less than 1 km from public transport | n.a. | 42.0 |  |
| HF has beds for overnight stay or routinely admits patients | 39.5 | 45.1 | 0.198 |
| *Index of readiness to deliver basic emergency obstetric and newborn care* (b) |  |  |  |
| HF has guidelines/protocols/educational materials for delivery | 35.8 | 39.1 | 0.619 |
| HF has guidelines/protocols/educational materials for partograph | 25.8 | 28.6 | 0.645 |
| HF staff ever received in-service training on care during labor and delivery | 50.8 | 57.1 | 0.344 |
| HF staff ever received in-service training on use of partograph | 28.3 | 32.4 | 0.510 |
| HF staff ever received in-service training on life-saving skills/emergency complications | 39.2 | 41.0 | 0.785 |
| HF staff ever received in-service training on management of high risk pregnancies | 38.3 | 55.2 | 0.011 |
| HF staff ever received in-service training on neonatal resuscitation | 43.3 | 51.4 | 0.225 |
| HF staff ever received in-service training on mother-to-child transmission of HIV/AIDS | 46.7 | 56.2 | 0.154 |
| HF staff ever received in-service training on exclusive breastfeeding | 55.8 | 61.0 | 0.437 |

Appendix Table 1 continued

| HF identified emergency transport as most important issue to address in delivery and newborn care | 1.7 | 2.9 | 0.546 |
| --- | --- | --- | --- |
| HF has 24-hour functioning light source (including lantern) for delivery | 66.7 | 61.9 | 0.457 |
| HF has mucous extractor for delivery | 57.8 | 60.0 | 0.704 |
| HF has manual vacuum aspirator for removing retained products of conception | 30.8 | 29.5 | 0.831 |
| HF has dilation and curettage kit for removing retained products of conception | 29.2 | 29.5 | 0.953 |
| HF has newborn bag/tube and mask | 36.7 | 35.2 | 0.824 |
| HF has clean gloves for delivery | 75.8 | 82.9 | 0.196 |
| HF has injectable uterotonic (i.e., Ergometrine) | 74.2 | 77.1 | 0.604 |
| HF observed to have injectable antibiotic – Bezathine Benzyl Penicillin Injection | 21.0 | 23.8 | 0.456 |
| HF observed to have injectable antibiotic – Benzyl Penicillin (Procaine) Injection (IM/IV) | 29.6 | 30.8 | 0.776 |
| HF observed to have injectable antibiotic – Streptomycin Injection | 21.0 | 22.0 | 0.783 |
| HF observed to have any injectable antibiotic (d) | 33.5 | 36.7 | 0.443 |
| HF observed to have intravenous solution – Normal saline | 30.0 | 26.6 | 0.382 |
| HF observed to have intravenous solution – Dextrose and saline | 30.5 | 26.9 | 0.373 |
| HF observed to have intravenous solution - Ringers Lactate | 12.0 | 15.0 | 0.320 |
| HF has intravenous infusion set | 35.6 | 27.6 | 0.050 |
| *Index of management practices supportive of quality maternal health services* |  |  |  |
| 1. Quality assurance (a) |  |  |  |
| HF uses supervisory checklist for health system components based on standards and protocol (e.g., service-specific equipment, medicines, and records) | 22.3 | 28.0 | 0.141 |
| HF uses supervisory checklist for health service provision (e.g. observation checklist) based on standards and protocol | 21.9 | 29.4 | 0.053 |

Appendix Table 1 continued

| **Component** | **2005** | **2009** | **Significance** |
| --- | --- | --- | --- |
| HF uses quality assurance systems for identifying and addressing quality of care that is implemented by staff for specific service levels (i.e., not carried out facility wide) | 20.6 | 26.2 | 0.134 |
| HF uses facility-wide review of mortality | 17.2 | 22.7 | 0.117 |
| HF uses periodic audit of medical records or service registers | 28.3 | 29.7 | 0.728 |
| HF uses a quality assurance committee/team | 17.2 | 19.6 | 0.481 |
| HF uses the regional/district health management teams for quality assurance | 17.2 | 19.2 | 0.545 |
| HF uses other quality assurance method | 6.9 | 5.9 | 0.668 |
| 1. Systems for determining client opinion about the HF or services (a) |  |  |  |
| HF has suggestion box | 8.6 | 10.1 | 0.546 |
| HF has client survey forms | 3.0 | 5.6 | 0.154 |
| HF conducts client interviews | 12.5 | 12.9 | 0.867 |
| HF uses other method for client opinion | 6.0 | 7.7 | 0.453 |
| 1. ANC/PPC supervision content in the past 6 months (c) |  |  |  |
| Supervisor checked records/reports | 32. 5 | 39.7 | 0.223 |
| Supervisor observed health care provider’s work | 32.5 | 38.8 | 0.282 |
| Supervisor provided feedback on health care provider’s performance | 26.5 | 32.8 | 0.264 |
| Supervisor provided updates on administrative or technical issues related to health care provider’s work | 28.5 | 35.3 | 0.231 |
| Supervisor discussed problems health care provider may have encountered | 29.8 | 37.9 | 0.162 |
| Supervisor discussed job expectations | 26.5 | 34.4 | 0.158 |
| Other | 15.9 | 15.5 | 0.933 |
| 1. Delivery/newborn care supervision content in the past 6 months (b) |  |  |  |
| Supervisor checked records/reports | 31.7 | 31.4 | 0.969 |
| Supervisor observed health care provider’s work | 29.2 | 30.5 | 0.830 |
| Supervisor provided feedback on health care provider’s performance | 22.5 | 27.6 | 0.376 |

Appendix Table 1 continued

| Supervisor provided updates on administrative or technical issues related to health care provider’s work | 26.7 | 29.5 | 0.634 |
| --- | --- | --- | --- |
| Supervisor discussed problems health care provider may have encountered | 28.3 | 30.5 | 0.725 |
| Supervisor discussed job expectations | 24.2 | 27.2 | 0.555 |
| 1. Other | 11.7 | 10.5 | 0.777 |
| HF participates in regular reviews of maternal or newborn deaths or near miss deaths (c) |  |  | 0.005 |
| Yes, for mothers | 10.0 | 20.0 |  |
| Yes, for newborns | 15.0 | 2.9 |  |
| Yes, for both | 23.3 | 21.0 |  |
| No, does not participate | 51.7 | 56.2 |  |
| HF has up-to-date ANC registers (b) |  |  | 0.316 |
| No register | 32.5 | 30.2 |  |
| Register not seen | 27.8 | 20.7 |  |
| Register seen, most recent entry > 7 days ago | 4.6 | 3.5 |  |
| Register seen, most recent entry within past 7 days | 35.1 | 45.7 |  |
| HF has up-to date birth registers (b) |  |  | 0.379 |
| No register | 30.8 | 29.5 |  |
| Register not seen | 17.5 | 18.1 |  |
| Register seen, most recent entry > 7 days ago | 16.7 | 9.5 |  |
| Register seen, most recent entry within past 7 days | 35.0 | 42.9 |  |
| HF has skilled birth attendant (doctor, nurse or midwife) at the facility or on call 24 hours a day, including weekends, to provide delivery care (b, e) |  |  |  |
| None present | 40.0 | 34.3 | 0.003 |
| On call, sometimes primary/auxiliary-level provider | 12.5 | 5.7 |  |
| On call, always secondary/ higher-level provider | 0.0 | 10.5 |  |
| Present, sometimes primary-level/auxiliary provider | 5.8 | 8.6 |  |
| Present, always secondary/ higher-level provider | 41.7 | 41.0 |  |

Appendix Table 1 continued

| **Component** | **2005** | **2009** | **Significance** |
| --- | --- | --- | --- |
| No. of HFs providing ANC | 151 | 116 |  |
| No. of HFs providing delivery/newborn care | 120 | 105 |  |
| Total number of HFs surveyed | 233 | 286 |  |

na Data not available/collected.

1. Data pertain to all HFs
2. Tabulations restricted to HFs providing delivery/newborn care unless otherwise indicated. For computation of the index all other HFs were assigned the value “0.”
3. Data pertain to HFs that provided ANC; for computation of the index all other HFs were assigned the value “0.”
4. Not included in the computation of the index; data included pertained to specific supplies/medication
5. A secondary or higher level provider was defined as a doctor, nurse, or midwife.
